# The effects of oligomerization on *Saccharomyces cerevisiae *Mcm4/6/7 function

**DOI:** 10.1186/1471-2091-11-37

**Published:** 2010-09-22

**Authors:** Xiaoli Ma, Brent E Stead, Atoosa Rezvanpour, Megan J Davey

**Affiliations:** 1 Department of Biochemistry, Schulich School of Medicine and Dentistry, University of Western Ontario, London, Ontario N6A 5C1, Canada

## Abstract

**Background:**

Minichromosome maintenance proteins (Mcm) 2, 3, 4, 5, 6 and 7 are related by sequence and form a variety of complexes that unwind DNA, including Mcm4/6/7. A Mcm4/6/7 trimer forms one half of the Mcm2-7 hexameric ring and can be thought of as the catalytic core of Mcm2-7, the replicative helicase in eukaryotic cells. Oligomeric analysis of Mcm4/6/7 suggests that it forms a hexamer containing two Mcm4/6/7 trimers, however, under certain conditions trimeric Mcm4/6/7 has also been observed. The functional significance of the different Mcm4/6/7 oligomeric states has not been assessed. The results of such an assessment would have implications for studies of both Mcm4/6/7 and Mcm2-7.

**Results:**

Here, we show that *Saccharomyces cerevisiae *Mcm4/6/7 reconstituted from individual subunits exists in an equilibrium of oligomeric forms in which smaller oligomers predominate in the absence of ATP. In addition, we found that ATP, which is required for Mcm4/6/7 activity, shifts the equilibrium towards larger oligomers, likely hexamers of Mcm4/6/7. ATPγS and to a lesser extent ADP also shift the equilibrium towards hexamers. Study of Mcm4/6/7 complexes containing mutations that interfere with the formation of inter-subunit ATP sites (arginine finger mutants) indicates that full activity of Mcm4/6/7 requires all of its ATP sites, which are formed in a hexamer and not a trimer. In keeping with this observation, Mcm4/6/7 binds DNA as a hexamer.

**Conclusions:**

The minimal functional unit of Mcm4/6/7 is a hexamer. One of the roles of ATP binding by Mcm4/6/7 may be to stabilize formation of hexamers.

## Background

Helicases assume a variety of oligomeric forms from monomers and dimers to larger oligomers such as hexamers and dodecamers (double hexamers; [1]). The oligomeric form of a helicase is often important for its function. For example, hexameric helicases form ring structures that are thought to encircle single and/or double stranded DNA as part of their unwinding function [1-7]. Furthermore, the number of ring constituents is often important for function; *Methanobacter thermoautotrophicum *MCM, which is isolated as hexamers, heptamers or dodecamers, is thought to function as a hexamer [8]. In contrast, some helicases are functional in different oligomeric forms, such as the hepatitis C virus NS3 helicase, which functions as a monomer, dimer or larger oligomers [9].

The minichromosome maintenance proteins 2 through 7 (Mcm2-7) form a ring-shaped heterohexamer that is the replicative helicase in eukaryotic cells [10]. Each of Mcm2 through 7 is essential for the initiation and elongation stages of DNA replication (for a review, see [10]) and helicase activity has been detected not only with Mcm2-7, but also with complexes containing different combinations of Mcm proteins [2,11-15], including a heterohexamer comprised of Mcm4, Mcm6 and Mcm7 that was initially purified from HeLa cells [12]. The biological significance of these additional helicase complexes is not known. Interestingly, Mcm proteins are present in cells at copy numbers that are far in excess of what is expected for two replication forks per active origin [16-20]. It has been proposed that the "excess" Mcm subunits have a role in activating dormant origins during replicative stress in *Xenopus *and human cells [21-23]. In addition, Mcm subunits have been implicated in other cellular processes such as gene expression [24-27], and maintenance of genomic stability [28-30]. Mcms also interact with chromatin remodeling factors and histones [12,31-34].

Mcm4/6/7 forms one half of the Mcm2-7 hexamer ring and may be the catalytic core of the Mcm2-7 helicase [35,36]. Differences between Mcm4/6/7 and Mcm2-7 are used to investigate Mcm2-7 mechanisms. Functional Mcm4/6/7 has been isolated as both a hexamer and a trimer [12,14,37-39]. Electron microscopy studies indicate that Mcm4/6/7 hexamers are ring shaped [38,40]. The functional significance of these observations has not been explored.

Here we show that Mcm4/6/7 exists in an equilibrium of oligomeric states in which smaller oligomers predominate in the absence of nucleotide. ATP or ATPγS and to a lesser extent, ADP shifts the equilibrium towards formation of larger oligomers. Use of Mcm mutants that are impaired in the function of intersubunit ATP sites (arginine finger mutants) indicates that Mcm4/6/7 functions as a hexamer. Thus, one of the roles of ATP in Mcm4/6/7 function is to promote oligomerization required for Mcm4/6/7 activity.

## Results

### Reconstitution of Mcm4/6/7 by anion exchange chromatography

**Figure 1:**
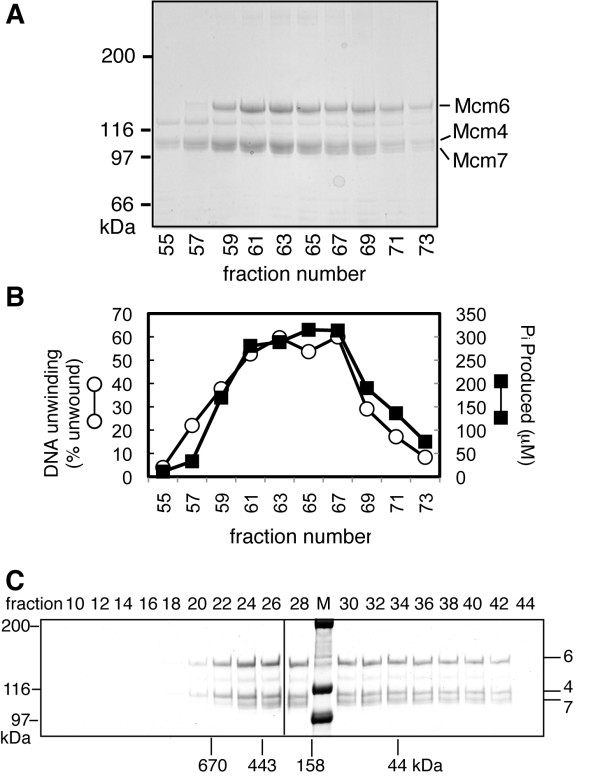
**Reconstitution of Mcm4/6/7 using anion exchange chromatography**. **A) **A portion of the indicated fractions from the Mono Q column were analyzed by SDS-PAGE (6%) and stained with Coomassie Brilliant Blue. The migration of size markers through the gel is indicated on the left. The migrations of Mcm4, Mcm6 and Mcm7 are indicated on the right. There is a minor contaminating band that migrates between Mcm6 and Mcm4 that is found in all of our preparations. **B) **ATP hydrolysis (black square) and DNA unwinding (white circle) by equal volumes of the indicated fractions were analyzed as described in the "Methods" section. **C) **A 50 μg portion of Mono Q fraction 67 was analyzed by gel filtration on a Superose 6 PC 3.2/30 column equilibrated in Buffer A with 100 mM NaCl. Shown here are Colloidal Coomassie Blue-stained SDS polyacrylamide gels (6%) of the indicated fractions. The vertical line marks the border between separate gels. The peak elutions of size standards are indicated at the bottom of the gel. The migration of size standards though the SDS gels is indicated on the left and of Mcm4, Mcm6 and Mcm7 on the right.

**Figure 2:**
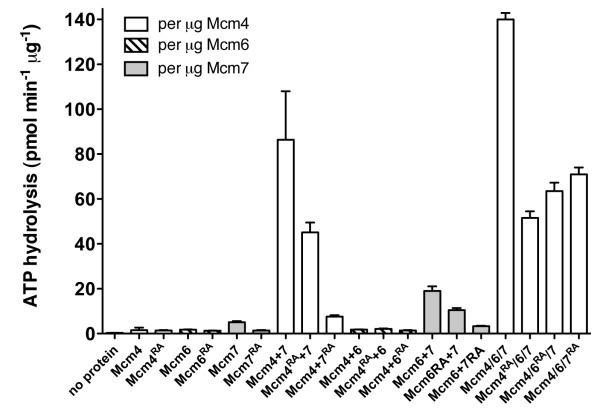
**ATP hydrolysis by individual and pairwise Mcms and Mcm4/6/7 complexes**. The mean rate of ATP hydrolysis for the indicated individual Mcms, pairwise combinations and Mcm4/6/7 complexes is shown. Rate measurements were determined at 1 mM ATP as described in "Methods". For the pairwise combinations of Mcm proteins, the rate is expressed per μg of the Mcm protein that is predicted to bind nucleotide within the pairs (as indicated). For comparison to Mcm4 + Mcm7, the rate of ATP hydrolysis by Mcm4/6/7 is expressed per μg Mcm4 (assuming equimolar ratios of each protein in the complex).

Mcm4/6/7 was reconstituted from individual subunits and isolated by anion exchange chromatograpy. A portion of each fraction was analyzed by Coomassie Brilliant Blue-stained SDS-PAGE to determine the presence of each protein (Figure 1A). In addition, the ability of each fraction to hydrolyze ATP or to unwind a synthetic fork substrate was determined (Figure 1B). Both ATPase activity and DNA unwinding activity co-eluted with fractions containing equal ratios of the three proteins. The lack of activity with individual Mcm subunits (Figure 2 and data not shown) and the activity associated with co-elution of all three subunits suggested that an active complex was formed.

We then examined Mcm4/6/7 by gel filtration analysis to determine the size of the active complex (Figure 1C). A peak fraction from the Mono Q elution containing equal ratios of Mcm4, Mcm6 and Mcm7 was analyzed by gel filtration. Comparison with size standards indicated that the elution of Mcm4/6/7 peaked around fraction 26, corresponding to an apparent molecular size of approximately 300 kDa, close to the predicted molecular size of trimer containing one of each subunit (315 kDa). Consistent with a trimer, densitometric analysis indicated that the peak fractions contained equal ratios of Mcm6, Mcm7 and Mcm4. Most importantly, it was clear that the peak of Mcm4/6/7, although fairly broad, did not elute as a hexamer (fraction 22 corresponds to hexamer, predicted size 630 kDa). We were concerned that the dilution of Mcm4/6/7 before and during gel filtration may have resulted in the dissociation of Mcm4/6/7 hexamers. Consistent with this idea, the Mcm4/6/7 elution trails off into later fractions, suggesting a disruption of the complex into single subunits. Thus, gel filtration analysis was repeated at a 5-fold higher protein concentration.

**Figure 3:**
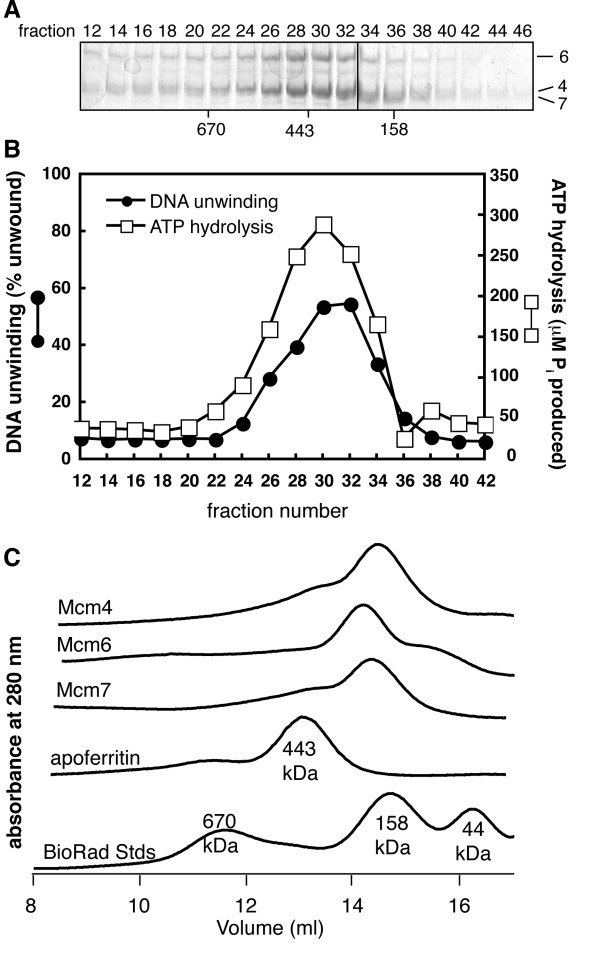
**Isolation of reconstituted Mcm4/6/7 by size exclusion chromatography**. **A) **A typical elution profile of Mcm4/6/7 from a Superose 6 10/300 GL column. A portion of the indicated fractions was analyzed by Coomassie Brilliant Blue-stained SDS-PAGE (6%). The vertical line marks the border between separate gels. The peak elution of size standards is indicated. The migration of size standards through the SDS gels is indicated on the left and of Mcm4, Mcm6 and Mcm7 on the right. **B) **Equal volumes of the indicated fractions were analyzed for ATP hydrolysis (white square) or DNA unwinding (black circle) as described in "Methods". **C) **The absorbance at 280 nm (A_280_) corresponding to the elution of each of Mcm4, Mcm6 and Mcm7 from the gel filtration column are shown. The peaks in absorbance correspond to the peak in the indicated proteins as determined by SDS-PAGE (not shown). The A_280 _curves of size standards (apoferritin, 443 kDa; BioRad standards which include thyroglobulin, 670 kDa; bovine gamma-globulin, 158 kDa and chicken ovalbumin, 44 kDa) are also shown.

Approximately 5 nmol each of Mcm4, Mcm6 and Mcm7 were mixed and then applied to a gel filtration column. A portion of each fraction was analyzed by Coomassie-stained SDS-PAGE as well as examined for DNA unwinding and ATP hydrolysis (Figure 3A and 3B). The resulting complex was active and the peak of activity co-eluted with the peak containing all three proteins and eluted earlier from the gel filtration column than the individual proteins (Figure 3C). Together, these observations suggest that Mcm4/6/7 formed and is active for DNA unwinding and ATP hydrolysis. The peak elution of Mcm4/6/7 (fr 29) corresponds to a size of approximately 330 kDa, the predicted size of a trimer. Thus, even at the increased protein concentration used for this experiment, the peak of active Mcm4/6/7 eluted at a position inconsistent with a hexamer.

### Adenine nucleotides affect the oligomerization of Mcm4/6/7

**Figure 4:**
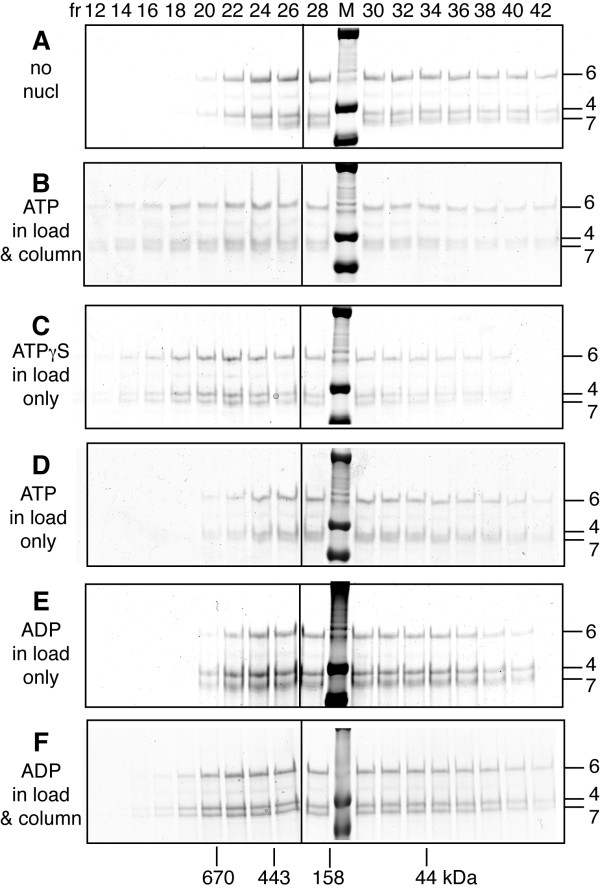
**Effect of different nucleotides on oligomerization of Mcm4/6/7**. The elution of Mcm4/6/7 from a 2.4 ml "mini" gel filtration column (Superose 6 PC 3.2/30; GE Healthcare) in the presence and absence of 5 mM adenine nucleotides is shown. A portion of the indicated fractions (above top panel) from each experiment was analyzed by GelCode Blue (Pierce) stained SDS-PAGE (6%). The migrations of Mcm4, Mcm6 and Mcm7 through the SDS gels are indicated to the right of each panel. Lanes containing molecular size markers are indicated (M) and their sizes are indicated on the left. The peak elution fractions of size standards (analyzed separately) are indicated below the bottom panel. The vertical lines mark the divisions between gels. In some experiments, nucleotide was added only to the protein (indicated by "load") and in some experiments nucleotide was added to both the protein and to the buffer used to equilibrate the gel filtration column ("load & column"). **A) **No nucleotide in either column buffer or load; **B) **5 mM ATP in column buffer and load; **C) **5 mM ATPγS added to load only; **D) **5 mM ATP added to load only; **E) **5 mM ADP in load only; and **F) **5 mM ADP in column buffer and load. UV profiles are not shown since addition of nucleotide interfered with the signal from protein.

The oligomerization of helicases is often affected by the nucleotides to which they bind [41-44]. To test whether ATP promotes oligomerization of *S. cerevisiae *Mcm4/6/7, we examined the effects of ATP, ATPγS and ADP on the oligomerization of Mcm4/6/7 using gel filtration. For these studies we used Mcm4/6/7 pre-formed in the absence of ATP. For comparison, the elution of Mcm4/6/7 from the gel filtration column in the absence of nucleotides from Figure 1 is shown (Figure 4A). Addition of ATP to both the column buffer and the protein applied to the column promoted an increase in the apparent size of Mcm4/6/7 (Figure 4B). The elution of Mcm4/6/7 with ATP is fairly broad, however the elution profile has shifted into earlier fractions than in the absence of nucleotide, suggesting an increase in size. To examine whether ATP hydrolysis by Mcm4/6/7 affects its oligomeric state, the effects of ATPγS and ADP were examined. ATPγS also supported elution of Mcm4/6/7 at an earlier volume than in the absence of nucleotide (~660 kDa; Figure 4C). It should be noted that ATPγS was only added to the protein and not to the column buffer in this experiment. For comparison, we performed an experiment in which ATP was only added to the protein and not the column buffer. Under these conditions, the peak of Mcm4/6/7 elution was similar to the elution in the absence of nucleotide (~440 kDa, fraction 25; Figure 4D). These results suggest that while ATP binding by Mcm4/6/7 promotes further oligomerization, production of ADP has a destabilizing influence on Mcm4/6/7 oligomers. To determine whether ADP supports a shift in Mcm4/6/7 elution volume, we examined the elution profile of Mcm4/6/7 with ADP present. When Mcm4/6/7 + ADP was analyzed through the gel filtration column equilibrated in nucleotide-free buffer, elution of Mcm4/6/7 closely resembled that of nucleotide free protein (~fraction 25; Figure 4E). When ADP was added to both the protein and the column equilibration buffer, there was a subtly and reproducibly different elution volume (Figure 4F). The intermediate size observed with ADP likely results from an equilibrium between different forms of Mcm4/6/7 that exchange more rapidly than they are separated through the gel filtration resin. Indeed, the ratio of Mcm6 to Mcm4/7 remains approximately 1:2 in the fractions from the ADP experiment, consistent with hexamer and trimer and not other intermediate size complexes (which would contain "extra" copies of one or two subunits). An exchange between different-sized complexes may also explain the relatively broad elution profile of Mcm4/6/7. Consistent with an equilibrium, the effect of ATP on Mcm4/6/7 was reversible; removal of ATP from Mcm4/6/7 hexamers and the buffer resulted in a return to elution of Mcm4/6/7 in the later fractions (data not shown).

Our observations are consistent with the idea that binding of ATP or ATPγS has a greater stabilizing effect on Mcm4/6/7 oligomerization than ADP. An alternative explanation is that ATPγS is bound better than ATP or ADP by Mcm4/6/7 and hence ATPγS more strongly promotes oligomerization. We discounted this possibility with the observation that ADP and ATPγS inhibit ATP hydrolysis by Mcm4/6/7 equally (data not shown), suggesting that ADP and ATPγS are bound equally but have different effects on oligomerization. These experiments suggested that ATP binding promotes oligomerization of Mcm4/6/7 and that ATP hydrolysis has a destabilizing influence.

### Chemical crosslinking of Mcm4/6/7

**Figure 5:**
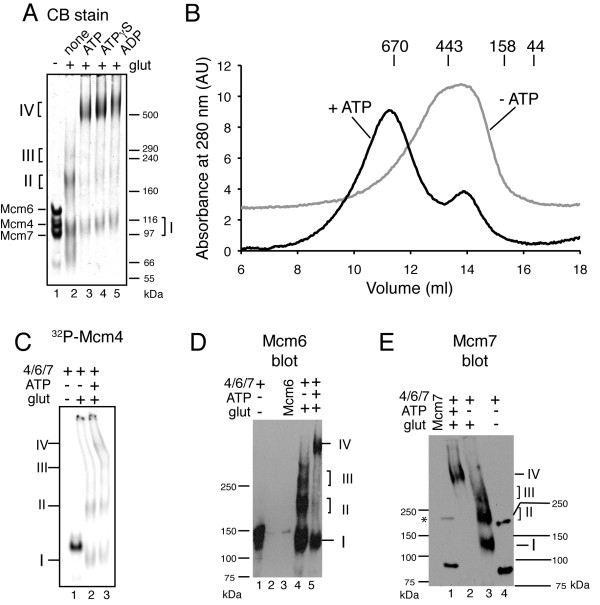
**Chemical crosslinking of Mcm4/6/7**. Crosslinking of Mcm4/6/7 with glutaraldehyde in the presence and absence of ATP, ADP or ATPγS is shown. **A) **The samples were analyzed on 3.5% SDS-phosphate gels stained with Coomassie Brilliant Blue ("CB stain"). Each lane contains 9 μg of Mcm4/6/7 in the presence and absence of glutaraldehyde and/or 1 mM ATP, ADP or ATPγS (as indicated). The migration of size standards is indicated on the right and of Mcm4, Mcm6 and Mcm7 in the absence of crosslinker is indicated on the left as are the migration of crosslinked products (by Roman numerals). **B) **Mcm4/6/7 was crosslinked with glutaraldehyde with and without 5 mM ATP before analysis on a Superose 6 10/300 GL column in the absence of added nucleotide. The A_280 _curves from the columns are shown for Mcm4/6/7 crosslinked in the presence of ATP (black line) and in the absence of ATP (grey line). The peak elution volumes of size standards, analyzed separately are shown above the graph. **C) **Phosphorimager scan of a SDS-phosphate gel of ^32^P-labeled Mcm4^PK^/6/7 crosslinked in the presence and absence of ATP (as indicated) is shown. The migrations of crosslinked products, based on Coomassie Blue staining of the same gel are indicated. Western blots to detect Mcm6 (**D**) and Mcm7 (**E**) in the crosslinked products. The migrations of Mcm6 and Mcm7 in the absence of crosslinker as well as the migration of crosslinked products are indicated. The presence of glutaraldehyde and ATP are indicated at the top of the blots. Standards with Mcm6 alone (40 ng) or Mcm7 alone (20 ng) are also indicated. The asterisk indicates a cross-reacting band with Mcm7 antibody that is barely visible in the Coomassie stained gels (see A).

To further explore the idea that ATP promotes oligomerization of Mcm4/6/7, we performed chemical crosslinking on Mcm4/6/7 with glutaraldehyde. When Mcm4/6/7 (isolated as "trimer" by gel filtration) was treated with glutaraldehyde in the absence of nucleotide and then analyzed on 3.5% acrylamide SDS-phosphate gels, a portion of Mcm4/6/7 was found in three diffuse bands, labeled "I", "II" and "III" in Figure 5A. Initially, we thought that band I corresponded to intramolecular crosslinked monomers, band II corresponded to dimers (predicted sizes ~ 200 kDa) and band III corresponded to trimers (predicted size 315 kDa) as that is most consistent with the migration of size standards on SDS-PAGE. However, when the products were analyzed by gel filtration, the elution volume was more consistent with a mixture of trimers and dimers rather than a mixture of monomers and dimers (Figure 5B), suggesting that band I corresponds to dimer and band II corresponds to trimers. Presumably, intramolecular crosslinks prevent complete unfolding of the proteins in SDS and result in a more compact protein with increased mobility through SDS-PAGE. Notably, a hexamer-sized band was not detected in the absence of nucleotide. In contrast, when 1 mM ATP, ATPγS or ADP was incubated with the same preparation of Mcm4/6/7 before treatment with glutaraldehyde, the majority of the protein shifts up to a band that migrates just above the 500 kDa marker on the SDS-phosphate gel, consistent with the nucleotides supporting the formation of larger oligomers (IV; Figure 5A). When the crosslinking reaction in the presence of ATP was analyzed by gel filtration (ATP was dialyzed away before gel filtration), Mcm4/6/7 eluted in two overlapping peaks (Figure 5B). The larger peak corresponded to a elution volume consistent with a hexamer whereas the second peak corresponded to a trimer-sized peak. SDS-phosphate gels of the gel filtration fractions confirmed that crosslinked hexamers (band IV) were present in the first peak and bands I, II and III were present in the second peak (data not shown). Addition of 0.1 mM or lower ATP, or 1 mM AMP did not support crosslinking of Mcm4/6/7 into the hexamer-sized band (data not shown). Use of antibodies against Mcm6 and Mcm7 as well as radiolabeled Mcm4 indicate that the crosslinked bands contain Mcm4, Mcm6 and Mcm7 (Figure 5C, D and 5E). These observations are consistent with the idea that binding of adenine nucleotides to Mcm4/6/7 promotes its oligomerization. In contrast to the gel filtration experiments in Figure 4, no differences were observed between ATP, ATPγS and ADP with the crosslinking. It is important to note that crosslinking disrupts equilibria and in the experiments shown here, hexamers are trapped by crosslinking. A similar disruption of equilibria by crosslinking has been observed previously [45]. Thus, we conclude that under the conditions utilized here, Mcm4/6/7 exists in an equilibrium of different oligomers. In the absence of nucleotide, smaller oligomers predominate whereas in the presence of ATP or ATPγS, the equilibrium shifts towards larger oligomers, likely hexamers.

### Mcm4/6/7 functions as a hexamer

**Figure 6:**
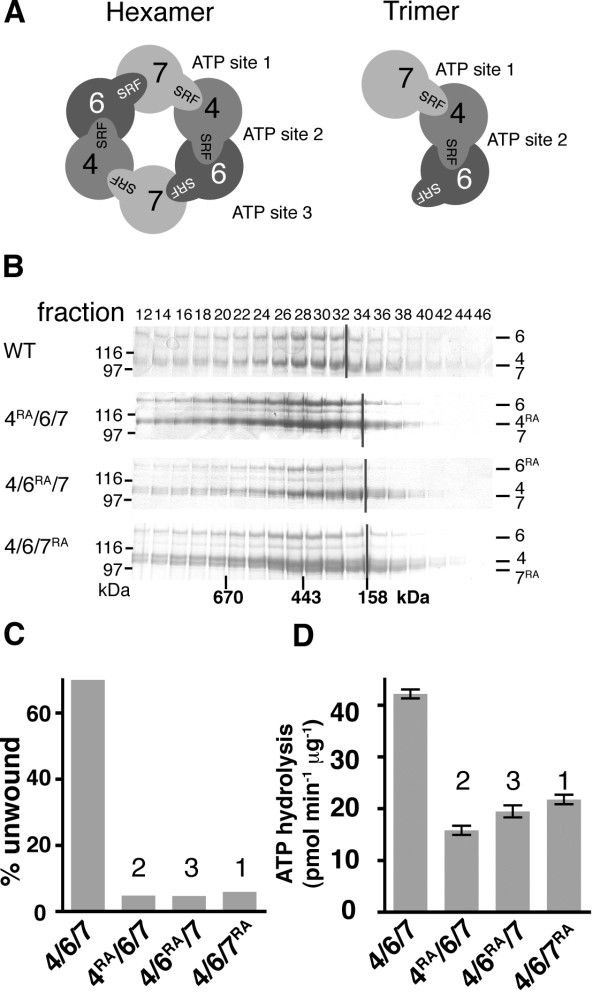
**Isolation and activity of Mcm4/6/7 complexes containing mutant proteins**. **A) **Models for the subunit arrangement in a Mcm4/6/7 hexamer (*left) *and trimer (*right*) are depicted. The complexes are drawn viewing the C-terminal face of the ring and are based on known physical interactions between *S. cerevisiae *MCM proteins (modified from [35]). "SRF" indicates the conserved motif encoding the arginine finger. Other arrangements of the Mcm4/6/7 hexamer are formally possible, however these alternatives are less likely than the model shown given the current knowledge of Mcm and related protein structures and the known physical interactions between Mcm proteins [10,14,35,47,61]. **B) **Mcm4/6/7 complexes were reconstituted using all wild-type proteins ('WT'), Mcm4^R701A^, Mcm6 and Mcm7 ("4^RA^/6/7"); Mcm4, Mcm6^R703A ^and Mcm7 ("4/6^RA^/7"); and Mcm4, Mcm6 and Mcm7^R593A ^("4/6/7^RA^") and the complexes were isolated by size exclusion chromatography as described in "Methods". The "WT" experiment shown here for comparison is from Figure 2. A portion of the indicated fractions was visualized by Coomassie Brilliant Blue stained SDS-PAGE (6%) to determine the peak fractions. The migrations of Mcm4, Mcm6 and Mcm7 (mutant or wild-type) through the gels are indicated to the right of each panel. The peak elution fractions of size standards analyzed separately are indicated below the bottom panel. **C) **DNA unwinding by wild-type and mutant Mcm4/6/7 complexes was compared. Each assay contained 750 ng of Mcm4/6/7. The numbers above the bars in the graph correspond to the ATP sites in part A. **D) **ATP hydrolysis was determined as described in "Methods". The mean rate per μg of Mcm4/6/7 is shown. The numbers above the bars in the graph correspond to the ATP sites shown in the hexamer model in part A and indicate which site is affected by the mutation.

The strongest evidence to date that Mcm4/6/7 functions as a hexamer is the finding that Mcm2, which inhibits DNA unwinding by Mcm4/6/7 disrupts Mcm4/6/7 hexamers to form an inactive Mcm2/4/6/7 tetramer [12]. With the observation that Mcm2 required nucleotide to inhibit Mcm4/6/7 but not to interact with it [46] as well as the observation here that active Mcm4/6/7 does not elute from gel filtration columns as a hexamer, the question of whether Mcm4/6/7 functions as a hexamer needs to be readdressed. We used mutational analysis, making use of the observations that ATP sites are formed at the interface of Mcm subunits and residues from both subunits are required for activity [35,47]. Specifically, one subunit contributes an arginine residue (often referred to as an arginine finger) for catalysis of ATP bound by the neighbouring subunit [35]. Mutation of the arginine fingers has been previously shown to interfere with ATP hydrolysis but not subunit interaction of Mcm proteins [35,47], thus mutations in the arginine fingers are ideally suited to test the number of subunit interfaces required for full activity of Mcm4/6/7 (Figure 6A). If Mcm4/6/7 is a trimer, mutation of only two of the three arginine fingers to alanine will affect Mcm4/6/7 activity. The third arginine (Mcm6 in the model in Figure 6A) is not expected to affect activity since it is not engaged in an ATP site. In contrast, if Mcm4/6/7 is a hexamer then mutation of each of the arginine fingers would affect function.

The arginine finger mutants Mcm4^R701A^, Mcm6^R708A ^and Mcm7^R593A ^were reconstituted with the corresponding wild-type proteins to yield Mcm4^RA^/6/7, Mcm4/6^RA^/7 and Mcm4/6/7^RA^, respectively (Figure 6B). Next, the ATPase and DNA unwinding activities of the mutant complexes were compared to wild-type complex using the peak fractions from each of the columns. We first examined our results to determine whether the mutant complexes had formed. We noted that the elution of the mutant Mcm4/6/7 complexes from the gel filtration column was closely similar to that of wild-type complex and differed from the individual proteins (Figure 6B and data not shown). In addition, ATPase activity was detected with the mutant complexes, albeit at a lower rate than wild-type complex (Figures 2 and 6D). This lower activity did not appear to be due to contaminating activity from pair-wise combinations of Mcm proteins. Our conclusion is based on the observations that the pairwise combinations of Mcm6 and 7 and Mcm4 and 6 have little or no ATPase activity compared to Mcm4/6/7; and that the extent to which mutation of Mcm4 and Mcm7 affect ATP hydrolysis differs between the pair-wise complexes (52% and 10% of wild type, respectively) and Mcm4/6/7 complexes (37% and 50% of wild type; Figure 2). We conclude from these results that the mutant Mcm4/6/7 complexes had formed.

With the assurance that the mutant complexes had formed, we then examined the results to determine whether Mcm4/6/7 functions as a trimer. DNA unwinding above background was not detected with Mcm4^RA^/6/7, Mcm4/6^RA^/7 or Mcm4/6/7^RA^, even at a concentration of protein at which wild-type complex unwinds approximately 70% of the substrate (Figure 6C). ATP hydrolysis was about 35-50% of that by non-mutant complex (Figure 6D). These results support the idea that Mcm4/6/7 functions as a hexamer since all three arginine fingers and hence the formation of interfaces between each of the proteins is required for full function of Mcm4/6/7.

### Mcm4/6/7 binds DNA as a hexamer

**Figure 7:**
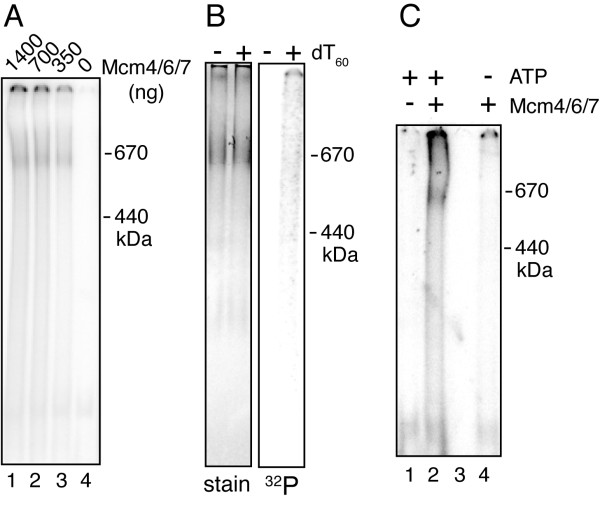
**Mcm4/6/7 binds single stranded DNA as a hexamer**. Mcm4/6/7 was crosslinked in the presence of single stranded DNA using glutaraldehyde. The indicated amounts of Mcm4/6/7 were incubated with 20 nM ^32^P-dT_60 _and 1 mM ATP in 6 μl of 20 mM HEPES NaOH pH 7.5 and 10 mM magnesium acetate. After incubation at 25°C for 10 min, 0.4 μl of 2.3% glutaraldehyde was added. The mixture was then incubated at 37°C for 2 min before quenching the reaction with 0.8 μl 2 M Tris. After addition of 4 μl 60% glycerol, the samples were analyzed on a 5% polyacrylamide gel in 0.5 × TBE. **A) **A phosphorimager scan of crosslinking of Mcm4/6/7 to dT_60 _in the presence of ATP. The migration of size markers, based on Coomassie Brilliant Blue staining of the gel is indicated on the right. Note that in order to resolve trimer and hexamer sized, the gel was run so that free DNA migrated off the bottom of the gel. **B**) Crosslinking of Mcm4/6/7 in the presence and absence of DNA (as indicated). A Phosphorimage is shown on the right and the same gel, stained with GelCode Blue (Pierce) is on the left. Migration of size standards is shown on the right. C). Crosslinking of Mcm4/6/7 in the presence and absence of 1 mM ATP, as indicated. Migration of size standards is shown on the right. Lane 3 is an empty lane.

If Mcm4/6/7 functions as a hexamer, then it should bind to DNA as a hexamer. To test this idea, we crosslinked Mcm4/6/7 bound to single stranded DNA using glutaraldehyde (Figure 7). To ensure single stranded DNA, we used polydeoxythymidine (dT_60_) as the substrate. Mcm4/6/7 was prepared in the absence of ATP and then incubated with DNA in the presence and absence of ATP. After crosslinking, the products were analyzed on a native gel along with size markers. In the presence of Mcm4/6/7 and crosslinker, the DNA migrated just below the thyroglobulin marker (670 kDa), consistent with the binding of hexamer to the DNA (Figure 7A). Staining of the gel with colloidal Coomassie Blue detected a single band at the same position as the DNA (Figure 7B). Furthermore, the migration of crosslinked Mcm4/6/7 through the gel is the same in the presence and absence of DNA (Figure 7B). The hexamer-sized band is protein dependent (Figure 7A, lane 4) and is dependent on ATP (Figure 7C). These experiments suggest that Mcm4/6/7 binds to single stranded DNA as a hexamer. Thus, the accumulated evidence in this study suggests that Mcm4/6/7 functions as a hexamer and that formation of the hexamer is supported by ATP.

## Discussion

We propose that Mcm4/6/7 exists in an equilibrium of oligomers that is shifted towards larger oligomers by ATP binding. Mcm4/6/7 that elutes from the gel filtration column as small oligomers, probably trimers, appears to be active. This is in seeming contrast to the mutagenic analysis indicating that all three types of ATP sites are required for Mcm4/6/7 function. It should be noted that the assays of Mcm4/6/7 function required the addition of ATP and this likely accounts for the activity of the smaller oligomers; ATP shifts the equilibrium towards larger oligomers, likely hexamers thus forming the active complex. These findings have implications for the use of Mcm4/6/7 as a probe for Mcm2-7 mechanisms.

### Mcm4/6/7 oligomerization

We suggest that Mcm4/6/7 oligomerizes into hexamers in the presence of ATP. Densitometry on the SDS-PAGE gels of the fractions from the gel filtration columns in the presence of ATP and ATPγS indicate that there are equal ratios of Mcm4, Mcm6 and Mcm7 and thus are consistent with hexamers, but not other sized complexes that would be expected to contain extra copies of one or two of the subunits. Furthermore, the hexameric form of Mcm4/6/7 is the form that is most frequently reported in other studies [12,14,38]. Here, the elution profiles from the gel filtration columns are relatively broad suggesting that there are different sized species. We suggest that Mcm4/6/7 exists in an equilibrium of different sized complexes that exchanges more rapidly than they are separated by gel filtration. We carried out additional biophysical measurements to assist in determining the size of the Mcm4/6/7 complex in the presence and absence of adenine nucleotides such as analytical ultracentrifugation and dynamic light scattering. While neither of these techniques was useful in determining a size, they both indicated that the size of Mcm4/6/7 increases when ATP was added (data not shown). Given the opposing influences of shape on gel filtration and sedimentation equilibrium ultracentrifugation as well as the dramatic increase in size in the cross-linking experiments, we concluded that the changes in elution in the gel filtration experiments dependent on ATP are not solely due to conformational changes rather than changes in oligomeric forms.

The observation of smaller oligomers in the absence of nucleotide is consistent with studies on mammalian Mcm4/6/7 expressed in insect cells where trimers have been observed under certain conditions [12,37,39], but contrasts with the observation of only hexamers with budding and fission yeast Mcm4/6/7 [14,38]. The strongest contrast is the ATP-dependence for oligomerization found in this study and the isolation of budding yeast Mcm4/6/7 hexamers by the Scwacha group [38]. There are many differences in the preparation of these complexes and a single factor that contributes to the differing oligomeric states cannot be identified. However, it is interesting to note that there are also differences in activity between Mcm4/6/7 isolated as a hexamer versus Mcm4/6/7 isolated as a trimer. Specifically, Mcm4/6/7 isolated as a trimer binds to circular single stranded DNA [46]. In contrast, with Mcm4/6/7 isolated as a hexamer, single stranded circular DNA was unable to compete Mcm4/6/7 away from linear oligonucleotides [11]. Consistent with this contrast, we have found that binding of Mcm4/6/7 to circular single stranded DNA is inhibited by first incubating Mcm4/6/7 in ATP or ATPγS, which promotes hexamerization (Additional File 1). One explanation of these findings is that Mcm4/6/7 hexamers are unable to bind to circular single stranded DNA because DNA is unable to access its binding sites in the interior of the ring. A similar effect has been observed with other helicases [44,48]. Note that crosslinking experiments suggest that Mcm4/6/7 bound to DNA is a hexamer (Figure 7). Thus, it is the formation of hexamer before binding to DNA and not formation of the hexamer *per se *that is inhibitory. Consistent with the lower DNA binding when incubated in ATP before DNA, Mcm4/6/7 reconstituted in the presence of ATP also had lower DNA unwinding activity (Additional File).

In addition to hexamers, there are also instances of Mcm complexes as double hexamers. Based on its DNA unwinding behaviour at "Y"-shaped forks, *S. pombe *Mcm4/6/7 is proposed to be more processive as a double hexamer. More recently, it was shown that *S. cerevisiae *Mcm2-7 assembles on origins as a double hexamer [49,50]. These observations are similar to double hexamers proposed for viral [48] and bacterial [51] replicative helicases. Formation of double hexamers of Mcm complexes likely requires the presence of DNA and/or origin assembly factors required for DNA replication initiation.

### ATP and the oligomerization of helicases

Nucleotides affect the oligomerization of other helicases such as bacteriophage T7 gene 4A [41], *E. coli *DnaB [42], *Bacillus subtilus *DnaC [44] and SV40 Tag [43]. Similar to Mcm proteins, the ATP sites of the aforementioned proteins are located at the interface of subunits and thus are ideally located to affect oligomerization. However, unlike Mcms these helicases are comprised of several copies of the same subunit. Thus, subunit interfaces differ only by the nucleotide binding state of the subunits involved. Conversely, Mcm4/6/7 not only has nucleotide-defined differences at its subunit interfaces, but also differences in interfaces defined by subunit composition (Figure 6A). Our model predicts that Mcm4/6/7 trimers would oligomerize into hexamers through interaction between Mcm6 and Mcm7. The predicted interaction between Mcm6 and Mcm7 may be the weak link in formation of Mcm4/6/7 hexamers. In our previous studies, we did not detect a pair-wise physical interaction between Mcm6 and Mcm7 under conditions where interactions of Mcm4 with Mcm6 or Mcm7 were detected [35]. In another study, co-expression of *S. cerevisiae *Mcm6 and Mcm7 in insect cells did not yield a stable complex [47]. Furthermore, we were unable to detect interaction between Mcm6 and Mcm7 in the presence of ATP and glutaraldehyde (unpublished), although a weak ATPase activity was noted when Mcm6 and Mcm7 were combined (Figure 2). In contrast, a synthetic lethal interaction between temperature sensitive alleles of genes encoding the *S. pombe *homologues of Mcm6 (*mis5-268*) and Mcm7 (*mcm7-98*) as well as a weak physical interaction between the human proteins have been reported [52,53]. It may be that a robust interaction between Mcm6 and Mcm7 requires Mcm4 in budding yeast. Alternatively, the relative weakness of a Mcm6-Mcm7 interaction may be compensated for by the presence of two Mcm6-7 interfaces in the Mcm4/6/7 hexamer.

### Mcm4/6/7 ATP site utilization

Mutation of the arginine fingers in Mcm4, Mcm6 or Mcm7 resulted in complexes that were unable to unwind DNA and had reduced ATPase activity. These observations suggested to us that Mcm4/6/7 does not function as a trimer. In addition, the effects of the arginine finger mutation on ATP hydrolysis by Mcm4/6/7 are not what one would expect if each of the ATP sites functioned independently of the others and contributed equally to ATP hydrolysis by the complex (67% of wild type). Rather, larger decreases in hydrolysis were observed, in keeping with the idea that ATP binding and/or hydrolysis at one site affects the activity of other subunits in the helicase ring. A similar effect has been observed in other helicases, including Mcm2-7 [47]. Indeed, Mcm complexes provide an excellent system to study this phenomenon since mutation of one or two subunits with defined positions in the helicase ring is more easily achieved in a heterohexamer than a homohexamer.

The contribution that each ATP site makes to the activity of the complex appears to be roughly equal (about 35-50% of wild type ATPase activity and all interfered with DNA unwinding). This observation contrasts to the studies of You and colleagues on mouse Mcm4/6/7, which indicated that each ATP site made different contributions to ATP hydrolysis, DNA binding and DNA unwinding [54]. The mouse study used mutations in the Walker A and B motifs, which in some proteins affects ATP binding as well as hydrolysis and this may account for the differences.

### Mcm4/6/7 within Mcm2-7

One of the motivations for studying Mcm4/6/7 is to discern differences between Mcm4/6/7 and Mcm2-7 that may yield clues to the mechanism of Mcm2-7. All of the subunits are essential for DNA unwinding *in vivo *[55]. In contrast, Mcm4/6/7 is sufficient for DNA unwinding *in vitro *and Mcm2 or Mcm3/5 inhibit DNA unwinding [12,14]. These observations have led to the suggestion that Mcm2, 3 and 5 may play essential regulatory roles within Mcm2-7. Recent mutational studies are consistent with that idea [11,46]. However, the proposal that only a subset of Mcm2-7 subunits participates directly in DNA unwinding must be reconciled with studies on homohexameric helicases in which all subunits participate directly in the molecular mechanisms required for DNA translocation and DNA unwinding [6,56]. Our conclusion that the active form of Mcm4/6/7 is the hexamer and not a trimer is a first step towards addressing this issue. Still to be addressed are the questions of whether the requirement of a hexameric Mcm4/6/7 is due to all six subunits participating directly in DNA unwinding or to formation of the ring, which tethers the helicase to DNA and/or forms a steric block for DNA unwinding.

## Conclusions

We conclude that *S. cerevisiae *Mcm4/6/7 exists in an equilibrium of different oligomers in solution. When ATP, ATPγS or ADP is added to Mcm4/6/7, the equilibrium shifts towards formation of larger oligomers. Finally, mutation of a residue important for the function of intersubunit ATP sites indicates that the three types of ATP site interfaces are required for full activity, consistent with Mcm4/6/7 functioning as a hexamer.

## Methods

### Materials

Sources of reagents were Sigma Aldrich for ATP, ADP and ATPγS (≥ 99% purity for each); New England BioLabs for molecular biology enzymes; UWO Oligo Factory for oligonucleotide primers and Integrated DNA Technologies for helicase substrates.

### Plasmids

Plasmids for the expression of Mcm4, Mcm6 and Mcm7 were described previously [35]. The SRF arginine (R701) of Mcm4 was changed to alanine using two PCR steps. In the first PCR step, a portion of the *MCM4 *ORF on pET11a-*MCM4 *was amplified using 5'd(CCACTACTTTCGGCATTCGATCTGGT)-3' and 5'-d(TAGTTATTCTCAGC)-3'. The product of the first PCR reaction was used as a megaprimer [57] in a second PCR reaction with 5'd(GTGAGCGGATAACAATTCCCCTCTAG) to amplify the entire *MCM4 *ORF from pET11a-*MCM4*. The resulting PCR product (2.9 kb) was digested with *Nde*I-*Blp*I and then inserted into the same sites of pET24a (Novagen) to generate pMD223.

An alanine substitution of arginine 708 in Mcm6 was generated by inverse PCR [58]. The primers, 5'-d(CAAAAAATAAATCAAATGCGGACATGATCGGTGCGG)-3' and d(TTATTCTTGATGACTGTAACG)-3' were phosphorylated using T4 polynucleotide kinase and then used to amplify pET16b-*MCM6*. After amplification, the parental template was digested with *Dpn*I and then circularized using T4 DNA ligase. The resulting plasmid (pMD226) contained *mcm6_R708A _*under control of the T7 RNA polymerase promoter.

The mutant genes were sequenced across the entire open reading frame (London Regional Genomics Center).

### Proteins

Mcm6 was purified as described previously except that the EAH Sepharose step was omitted [35]. For ATPase assays, Mcm6 was further fractionated via gel filtration exclusion chromatography as previously described [35]. Mcm4^PK ^and Mcm4^PK^/6/7 were prepared as described previously [46]. The identities of the purified proteins were confirmed by Western blotting and/or MALDI mass spectrometry at the London Regional Proteomics Facility.

### Mcm4 purification

Mcm4 purification was a modification of a previous procedure [35]. After ammonium sulphate precipitation, protein was resuspended in 200 ml of Buffer B (20 mM HEPES-NaOH pH 7.5, 0.1 mM EDTA, 2 mM DTT and 10% (v/v) glycerol) and dialyzed against the same buffer for approximately 16 hours. The protein (Fr II; 5,752 mg) was applied to a 200 ml Fast Flow SP Sepharose column (GE Healthcare) equilibrated in Buffer B + 100 mM NaCl, washed with Buffer B and then eluted with a 2000 ml, 100 to 500 mM NaCl gradient in Buffer B. Peak fractions were pooled (FrIII; 210 ml, 147 mg), dialyzed against Buffer B + 100 mM NaCl and then applied to a 60 ml Heparin agarose (Bio-Rad) column equilibrated in Buffer B + 100 mM NaCl. The column was washed with 300 ml of the same buffer before elution with a 600 ml, 100 mM to 500 mM NaCl gradient in Buffer B. Peak fractions containing Mcm4 were pooled (Fr IV, 157 ml, 110 mg) and dialyzed against Buffer B and then 20 mg was applied to a 20 ml single stranded DNA (ssDNA) Sepharose column [59] equilibrated against Buffer B, washed with the same buffer and then eluted with a 200 ml, 0 to 500 mM NaCl gradient in Buffer B. The peak fractions were pooled (FrV, 23 ml, 14 mg) and then dialyzed against Buffer B before being applied to a 1 ml Mono S column equilibrated in Buffer B with 100 mM NaCl. The column was washed in Buffer B with 100 mM NaCl and then eluted with a 20 ml, 100 to 500 mM NaCl gradient in Buffer B.

### Mcm7 purification

Mcm7 and Mcm7^R593A ^were purified as described previously [35], except that additional purification steps were added to remove a contaminating nuclease activity. The pooled Mcm7 fractions from the Mono Q column ([35]; FrV, 7 ml, 8 mg) were dialyzed against Buffer B and then applied to a 1.7 ml Mono S column equilibrated in Buffer B + 100 mM NaCl. The column was washed with 5.5 ml Buffer B containing 100 mM NaCl. The majority of Mcm7 eluted from the column during the wash step as determined by Coomassie Brilliant Blue R 250 stained SDS-PAGE. The wash from the Mono S column (FrVI, 6 ml, 5 mg) was dialyzed in Buffer B before applying the protein to a 7 ml ssDNA Sepharose column equilibrated in Buffer B. The column was washed with 35 ml of the same buffer and bound proteins were eluted with a 70 ml, 0 to 500 mM NaCl gradient in Buffer B.

### Reconstitution of Mcm4/6/7 by anion exchange chromatography

Equimolar ratios (45 nmol) of Mcm6, Mcm4 and Mcm7 were mixed and then concentrated and desalted in a centrifugal ultrafiltration device (MWCO = 50 kDa) to approximately 15 mg/ml total protein concentration and a conductivity equivalent to 50 mM NaCl in Buffer A. The mixture was incubated at 16°C for 30 min before centrifugation in a table top centrifuge for 10 min at 10,000 × g. The sample was then applied to a 1 ml MonoQ column equilibrated in Buffer A + 50 mM NaCl, washed with 5 ml of the same buffer and then the protein was eluted using a 20 ml, 50 mM to 500 mM linear NaCl gradient in Buffer A. Mcm4/6/7 typically elutes at ~350-400 mM NaCl. Peak fractions, determined by Coomassie Blue R250-stained gels and enzymatic assays, were stored at -80°C.

### Mini-gel filtration of Mcm4/6/7

Mcm4/6/7 from the MonoQ column was analyzed on a 2.4 ml "mini" gel filtration column (Superose 6 PC 3.2/30; GE Healthcare) in the presence and absence of 1 mM adenine nucleotides (as indicated). Fifty μg of Mcm4/6/7 from the MonoQ column was incubated with or without 1 mM nucleotide and 10 mM Mg-acetate in 50 μl at 16°C for 30 minutes before centrifugation for 10 minutes at top speed in a table top centrifuge. The supernatant was then applied to the column and a void volume of 0.9 ml was collected followed by fractions of 50 μl each.

### Gel filtration reconstitution of Mcm4/6/7

Mcm4, Mcm6 and Mcm7 were combined (5.2 nmol of each) and then de-salted and concentrated to 5 mg/ml using an Amicon Ultra ultrafiltration device (MWCO = 50; Whatman). The mixture was incubated at 16°C for 30 min before centrifugation at 15, 000 × g for 10 min. The supernatant was decanted and immediately applied to a Superose 6 10/300 GL column (GE Healthcare) equilibrated in 20 mM Tris-HCl pH 7.5, 0.1 mM EDTA, 2 mM DTT, 100 mM NaCl and 10% (v/v) glycerol. After a void volume of 5.9 ml, 80 fractions of 250 μl each were collected. Twenty μl of the peak fractions were analyzed by SDS-PAGE (6%) stained with Coomassie Brilliant Blue R-250.

### Activity assays

ATP hydrolysis and DNA unwinding was measured as previously described [46]. For ATPase assay of pre-formed complexes, 1.25 μg of protein was used and for assay of single and pair-wise combinations, 0.5 μM (as monomer) of each protein was used in a total volume of 20 μl. DNA unwinding assays used the amounts of proteins indicated in the figure legends.

### Chemical crosslinking

Mcm4/6/7 was crosslinked with glutaraldehyde in the presence and absence of adenine nucleotides. Each reaction contained 9 μg Mcm4/6/7 in 23.7 μl of 20 mM HEPES NaOH pH 8.4, 10 mM magnesium acetate and 1 mM of the indicated nucleotide. The mixture was incubated at 16°C for 30 min before addition of 1.3 μl of 2.3% glutaraldehyde (EM grade, Polysciences Inc., Warrington, PA) followed by incubation for 2 min at 37°C. The reaction was quenched with 2.5 μl of 1 M Tris. Samples were analyzed on 3.5% acrylamide gels using a SDS-phosphate buffer system [60]. For visualisation of Mcm4, Mcm4^PK^/6/7 radiolabed with ^32^P [46] was substituted. For Westerns, only one tenth of the sample was analyzed. For gel filtration analysis 260 μg of Mcm4/6/7 in 520 μl was analyzed and the volumes of reagents were increased proportionally. After quenching, the sample was dialyzed against Buffer A (20 mM Tris-HCl pH 7.5, 0.1 mM EDTA, 2 mM DTT and 10% (v/v) glycerol) with 100 mM NaCl and then concentrated using an Ultrafree centrifugal filter device (50 K, Millipore) to 200 μl. The sample was then applied to a Superose 6 10/300 GL column and treated as described for uncrosslinked Mcm4/6/7 in the previous section.

### Western blotting

After separation by SDS-phosphate PAGE, the proteins were transferred to PVDF. The membranes were blocked for 15-20 hr at 4°C in casein blocking buffer (Sigma-Aldrich). All subsequent washes and incubations were performed at room temperature using TBS-T (20 mM Tris-HCl pH 7.6, 137 mM NaCl and 0.1% (v/v) polyoxyethylene (20) sorbitan monolaurate). The membranes were washed in 20 ml of TBS-T once for 15 min and twice for 5 min before incubation with either anti-Mcm7 antibody (1:10, 000, Santa Cruz Biotechnology Inc., sc6688) or with His-Probe-HRP reagent (1:500, Thermo Scientific) for 2 h. After washing, the Mcm7 blot was incubated with 1:10, 000 rabbit anti-goat immunoglobulin coupled to horseradish peroxidase (Sigma-Aldrich), before washing the blot again. The His-Probe-HRP blot was washed and both blots were developed for chemiluminesence using the manufacturer's instructions (Thermo Scientific).

## Authors' contributions

XM carried out experiments in Figure 2, 3, 4, 5, 6, 7 and S2 and participated in the design of experiments, BES carried out experiments in Figures 1 and S1 and biophysical studies on Mcm4/6/7 and participated in the design, conception and editing of the manuscript, AR created mutant proteins and carried out initial experiments for portions of Figure 2, MJD conceived of and assisted in execution of the study and wrote the manuscript. All authors read and approved the final manuscript.

## Supplementary Material

Additional file 1 **Additional Data for Ma et al**. File contains DNA binding and DNA unwinding experiments by Mcm4/6/7 pre-incubated or assembled in ATP. Click here for file
