# Supplementary material for: The effects of oligomerization on Saccharomyces cerevisiae Mcm4/6/7 function
Source: BMC Biochem. 2010 Sep 22;11:37. doi: 10.1186/1471-2091-11-37 (PMC2949612; doi:10.1186/1471-2091-11-37)
Supplement: Additional file 1 — Additional Data for Ma et al. File contains DNA binding and DNA unwinding experiments by Mcm4/6/7 pre-incubated or assembled in ATP. [file 1471-2091-11-37-S1.PDF]

SUPPLEMENTAL INFORMATION FOR MA et al

Effects of oligomerization on DNA binding

We examined Mcm4/6/7 binding in a gel filtration-based assay [1] using circular single stranded DNA (ssDNA). Mcm4/6/7 was radioactively labeled via a Protein Kinase A (PKA) site fused to Mcm4. The addition of the PKA tag does not interfere with Mcm4/6/7 function [1]. The DNA binding mixtures were applied to a gel filtration column from which the DNA substrate, along with any protein bound to it, eluted early from the column (fr 10-14) whereas free protein eluted in later fractions (fr 15-30). To determine whether the oligomerization of Mcm4/6/7 promoted by the addition of nucleotide affects binding to circular DNA, we tested whether an initial incubation with nucleotide altered the extent of Mcm4/6/7 binding to DNA. Note that *S. cerevisiae* Mcm4/6/7 is dependent on nucleotide for binding to ssDNA [1, 2]. When Mcm4/6/7 was incubated with DNA before ATP, there was a relatively large peak in the early fractions corresponding to Mcm4/6/7 bound to ssDNA (circles, Figure S1A). When Mcm4/6/7 was incubated with ATP before adding ssDNA, Mcm4/6/7 binding to DNA was only 67 ± 2.2 % that of binding when ATP was added after DNA (squares, Figure S1A). One explanation for this observation is that oligomerization of Mcm4/6/7 (likely as a ring, based on previous studies [2, 3]), blocks access of ssDNA to its binding sites on the interior of the ring. We further tested this model by using ADP, which weakly stabilizes hexamers and ATPγS, which has a stronger effect, in place of ATP. When ATPγS was included in the first incubation, there was a decrease in binding to ssDNA (60 ± 0.5 % that of binding when ATPγS was added after DNA; Figure S1B). In contrast,

preincubation with ADP had only a modest effect on DNA binding, resulting in a decrease in binding to 89 ± 3.9% of DNA binding observed when ADP was added after DNA (Figure S1C), in keeping with ADP's modest effect on hexamerization of Mcm4/6/7. These observations are in keeping with the idea that nucleotide-induced oligomerization of Mcm4/6/7 blocks access of ssDNA to its binding sites on the interior of the protein ring. That ssDNA binding sites are found on the interior of the ring is consistent with other hexameric helicases including T7 gp4 and E1 helicase [4, 5].

DNA unwinding by Mcm4/6/7 reconstituted in the presence of ATP

We asked whether formation of Mcm4/6/7 in the presence of ATP (Mcm4/6/7^{ATP}) unwound DNA similarly to Mcm4/6/7 that was formed without the addition of nucleotide (Mcm4/6/7^{none}). We took advantage of an EcoRI site located in the duplex region of the helicase substrate that will only be cut on duplex DNA. Thus, DNA unwinding is detected by the accumulation of uncut DNA. Using this approach, we detected DNA unwinding by Mcm4/6/7^{none} (Figure S2). In contrast, we detected very little DNA unwinding activity with Mcm4/6/7^{ATP} (Figure S2). Despite the lower DNA unwinding, by Mcm4/6/7^{ATP}, it was still a functional ATPase with a rate comparable to Mcm4/6/7^{none} (43 pmol/μg Mcm4/6/7/min). These results suggest that the reconstitution of Mcm4/6/7 in the presence of ATP causes a change in the complex that interferes with DNA unwinding, but not ATP hydrolysis. Since ATP caused further oligomerization of Mcm4/6/7, it is likely that it is the oligomerization of Mcm4/6/7 that is interfering with its activity, perhaps the initial binding to DNA.

References

1. Stead BE, Sorbara CD, Brandl CJ, Davey MJ: **ATP Binding and hydrolysis by Mcm2 regulate DNA binding by Mcm complexes.** *Journal of Molecular Biology* 2009, **391:**301-313.
2. Bochman ML, Schwacha A: **Differences in the single-stranded DNA binding activities of MCM2-7 and MCM467: MCM2 and MCM5 define a slow ATP-dependent step.** *J Biol Chem* 2007, **282:**33795-33804.
3. Sato M, Gotow T, You Z, Komamura-Kohno Y, Uchiyama Y, Yabuta N, Nojima H, Ishimi Y: **Electron microscopic observation and single-stranded DNA binding activity of the Mcm4,6,7 complex.** *J Mol Biol* 2000, **300:**421-431.
4. Egelman EH, Yu X, Wild R, Hingorani MM, Patel SS: **Bacteriophage T7 helicase/primase proteins form rings around single-stranded DNA that suggest a general structure for hexameric helicases.** *Proceedings of the National Academy of Sciences of the United States of America* 1995, **92:**3869-3873.
5. Enemark EJ, Joshua-Tor L: **Mechanism of DNA translocation in a replicative hexameric helicase.** *Nature* 2006, **442:**270-275.

Figure S1 Effect of hexamerization on DNA binding

For DNA binding, 0.6 μg of ^{32}P-Mcm4^{PK}/6/7 (0.9 μCi/μg) was pre-incubated in 20 mM Tris-acetate pH 7.5, 10 mM magnesium acetate, 0.1 mM EDTA and 2 mM DTT alone or with 5 mM ATP (A), ATPγS (B) or ADP (C) before addition of 2 pmol of M13mp18 single stranded DNA or buffer (as indicated) in a total volume of 100 μl. After incubation for 10 min at 37ºC, the samples were applied to a 5 ml 4 % plain agarose (Agarose Bead Technologies) gel filtration column equilibrated in 20 mM Tris-HCl pH 7.5, 0.1 mM EDTA, 2 mM DTT, 100 mM NaCl, 10 mM magnesium acetate and 40 μg/ml BSA. The amount of Mcm4/6/7 in each fraction was determined by scintillation counting. The elution profiles of Mcm4^{PK}/6/7 incubated with nucleotide first (○) or with DNA first (■) are shown. Protein bound to DNA eluted in the early fractions (fr 10-14) whereas free protein eluted later (fr 15-30). Shown are representative elution profiles of at least three separate experiments. The elution volume of DNA was confirmed by agarose gel

electrophoresis and co-elution of Mcm6 and Mcm7 with DNA was confirmed by Western blotting for select experiments.

Figure S2 DNA unwinding by Mcm4/6/7 reconstituted in the presence of ATP

A) Mcm4/6/7 was reconstituted with or without ATP (as indicated) and its ability to unwind DNA was measured. Increasing amounts (as indicated) of protein were added to 1 nM helicase substrate in Assay buffer containing 5 mM creatine phosphate, 0.2 mg/ml creatine kinase and 5 mM ATP in a total volume of 6 µl. After incubation at 37ºC for 10 min, 1 µl of EcoRI (20 u/µl) was added. After further incubation for 5 min at 37ºC, the assays were quenched by the addition of proteinase K for 1 min at 37ºC followed by the addition of 8 µl of 50 mM EDTA. The DNA was extracted with phenol/chloroform, ethanol precipitated and resuspended in 98 % formamide containing 0.1 % bromophenol blue and 0.1 % xylene cyanol. After heating to 95ºC for 5 min, the samples were applied to an 8 % acrylamide gel containing 6 M urea. The gel was subjected to a current of 1800 V for 20 min, dried and then exposed to a PhosphorStorage screen. The migrations of uncut DNA (indicating ssDNA) and cleaved product (indicating dsDNA) through the gel are shown on the left. **B**) Quantification of the gel in A is shown. Percent DNA unwinding by Mcm4/6/7^{ATP} (•) and Mcm4/6/7^{none} (O) was determined from the ratio of the single stranded band to the sum of the single stranded and double stranded bands after subtracting a background value.

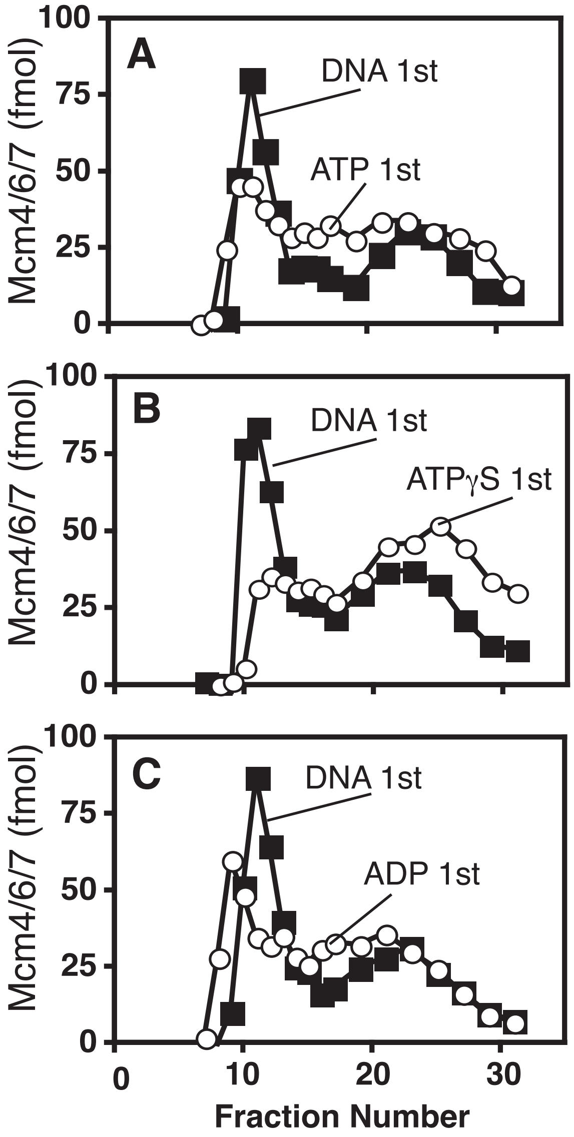

Ma et al Figure S1

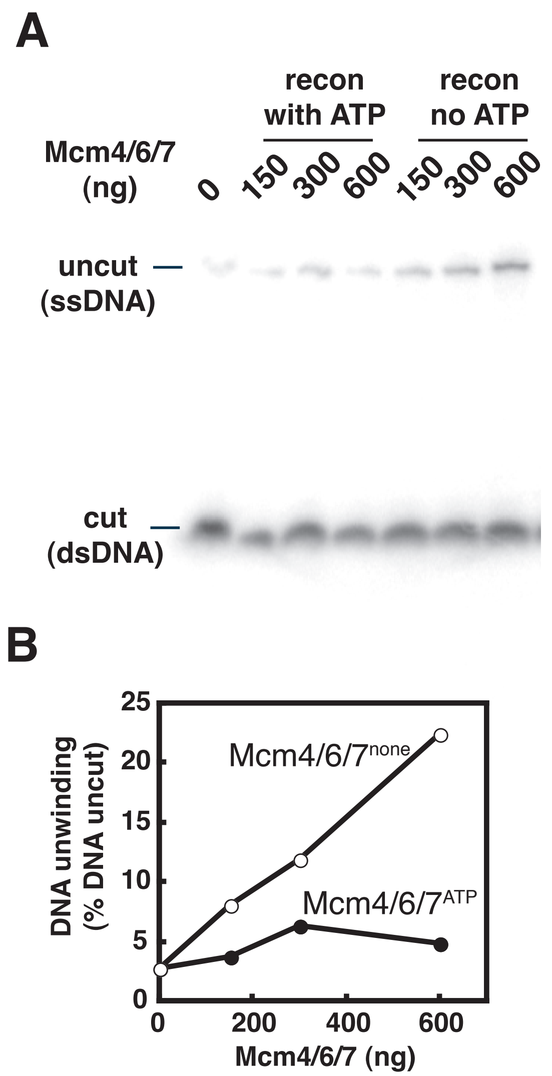

Ma et al Figure S2
